# Preparation and mechanical performance of fluorite tailings geopolymer precursor under alkaline heat activation

**DOI:** 10.1038/s41598-024-82560-y

**Published:** 2025-01-11

**Authors:** Hao Qiu, Hongtao Su, Hengyuan Liu, Zhijian Guo, Huifang Zhang, Juntao Ma, Xiao Wang

**Affiliations:** 1 School of Civil Engineering and Communication, North China University of Water Resources and Electric Power, Zhengzhou, 450045 People’s Republic of China; 2 Henan Transport Investment Group Pingwan Expressway Co., Ltd, Zhengzhou, 450016 China; 3 Zhongde Xinya Building Materials Co., Ltd, Zhengzhou, 450100 People’s Republic of China

**Keywords:** Fluorite tailings, Alkali heat activation, Geopolymer precursor, Amorphous glass phase, Engineering, Materials science

## Abstract

As one of the bulk solid wastes in the Yellow River basin in China, fluorite tailings urgently need to be utilized as resources. In this paper, NaOH and Na_2_CO_3_ were used for alkali thermal activation of ground fluorite tailings under different temperature conditions, and the reactivity was analyzed by XRD, SEM and compressive strength after hydration, so as to evaluate the feasibility of fluorite tailings as geopolymer precursor. The results show that the fluorite tailings can exhibit certain reactivity under alkaline heat excitation, and significant amorphous glass phase can be detected. The better heat excitation temperature is 1000 °C, while there is not enough amorphous glass phase for hydration reaction at the lower or higher temperature. The compressive strength of the tailings harden paste can reach 7.2 MPa at 28d after excitation with 50%NaOH at 1000 °C, which is expected to be used as geopolymer precursor after excitation.

## Introduction

**Fig. 1:**
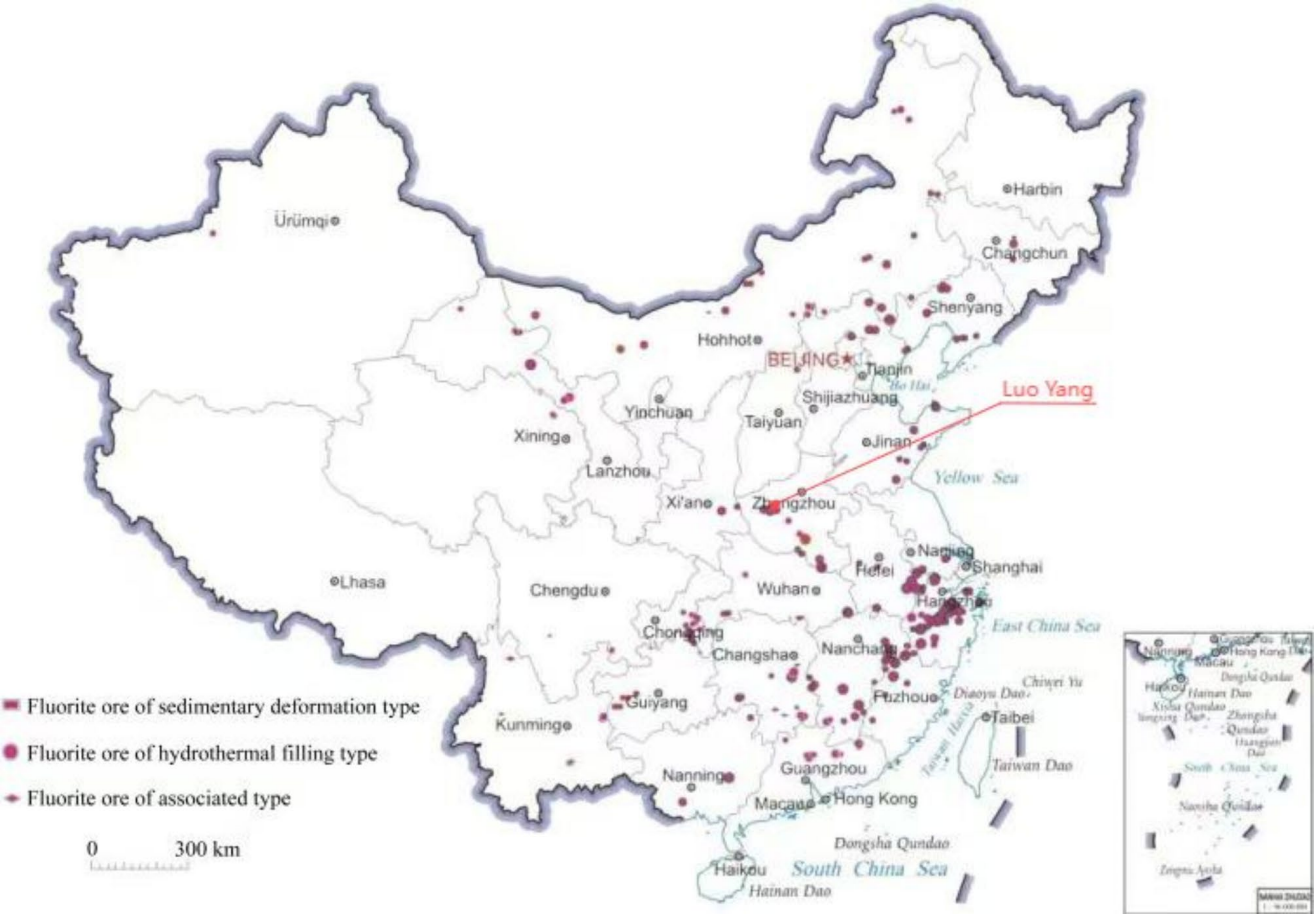
Distribution of fluorite deposits in China^1^.

A large amount of fluorite tailings is produced as a by-product of the industrial production of hydrofluoric acid. Fluorite ore in China is widely distributed as shown in Fig. 1^1^.At present, it is one of the bulk solid wastes, with a total reserve of more than 240 million tons^2^. If fluorite tailings fail to be handled properly, it may lead to water pollution, land occupation, and geological disasters. Therefore, fluorite tailings are urgently required for resource utilization^3,4^.

At present, most research on the resource utilization of fluorite tailings focuses on the secondary recovery of useful minerals and the preparation of microcrystalline glass^5–7^. Owing to their potential activity, most metallurgical tailings are used as precursors for activation and cementitious materials. Feng Rao and Qi Liu^8^ reviews the potential applications of geopolymer precursors in tailings excitation, which indicated that the alkali activating reaction is effective for the excitation and strengthening of the tailings containing aluminosilicates. The review shows that the alkali activation can stimulate and strengthen tailings effectively only when there is enough aluminosilicate in the local polymer reaction^9^ gold mine tailings mainly contain SiO_2_ and Al_2_O_3_, which can be used as aluminosilicate precursors of one component geopolymer. However, Ouffa^10^ pointed out that it is difficult to directly activate tailings through alkaline activation. Feng^11^ indicated that stable albiar could be transformed into glassy precursors by calcination at 850–1150 °C in the presence of an alkali source. Ke^12^ found that silicate and aluminosilicate phases from red mud were decomposed by alkali fusion at 800 °C to form a series of new phases, which were mainly disordered peralkaline aluminosilicate and calcium-rich phases. Moukannaa^13^ mixed phosphate tailings with NaOH and calcined them at temperatures from 550 °C to 800 °C, after which the main mineral phase structure changed significantly, and a new sodium-rich crystalline phase was formed. Quartz, a common impurity in some clay minerals, can be converted into an active amorphous phase^14^, suggesting that alkali melt-activated gold mine tailings can be used as precursors for one-component geopolymers. In addition, bentonite, kaolin, and halloysite calcined with alkali sources at high temperatures are suitable materials for the production of one-component geopolymers. The optimal temperature and alkali dose depend on the properties of the starting material^15–17^.

Geopolymer is a new type of inorganic material with excellent mechanical properties, low permeability, and high resistance to harsh environments commonly found in nature^18–22^. Geopolymers are synthesized by a series of solubility-recombination gel-hardening crystallization reactions between the raw materials and alkaline solutions^23^. Alkali melting can significantly destroy the crystal structure of the inert precursor, and active alkaline aluminum silicate glass is the main activation product^24,25^. Calcination temperature is an important factor affecting the pretreatment effect. Luo^26^ found that adding NaOH during calcination for 1.5 h at 600 °C could improve the Si solubility of vanadium tailings. Using NaOH as the base source, after calcination at 800–900  °C for 1 h, the crystalline structure of the straw stone, hydrobauxite, and garnet minerals in the red mud were destroyed, thus improving the reactivity^18,27^. The quartz phase in bentonite decomposes after activation by alkali melting at 700–1000 °C for 3 h^28^. Moukannaa^25^ found that in the presence of NaOH, the crystallinity of tailings decreased after calcination at 800 °C for 2 h. The alkali melting process requires high-temperature calcination, which increases the manufacturing cost of geopolymer samples. To solve this problem, an alkali hydrothermal technique can be used^29,30^. The crystallinity of tailings decreased significantly after alkali water heat treatment at 200 °C, and aluminosilicate glass was the main activated reactant. Dingwu Feng^11^ demonstrated the great potential of albitite as a geopolymer precursor by thermal activation of albitite using NaOH and Na_2_CO_3_, which provide a powerful support for the feasible of alkali heat activation.

The composition of fluorite tailings is similar to that of most tailings, but currently, there is no research on the activation of fluorite tailings. In this study, powdered fluorite tailings were used as the raw materials and combined with sodium hydroxide and sodium carbonate for high-temperature calcination. The activation effect is analyzed through XRD (X-ray Diffraction) diffraction analysis, FTIR (Fourier Transform Infrared) analysis, microscopic morphology observation by SEM (Scanning Electron Microscope) and EDS (Energy Dispersive Spectrometer). The feasibility of the alkali thermal activation treatment of fluorite tailings as a precursor for geopolymers was evaluated based on their mechanical properties, and their mechanism of action was analyzed.

## Materials

**Fig. 2:**
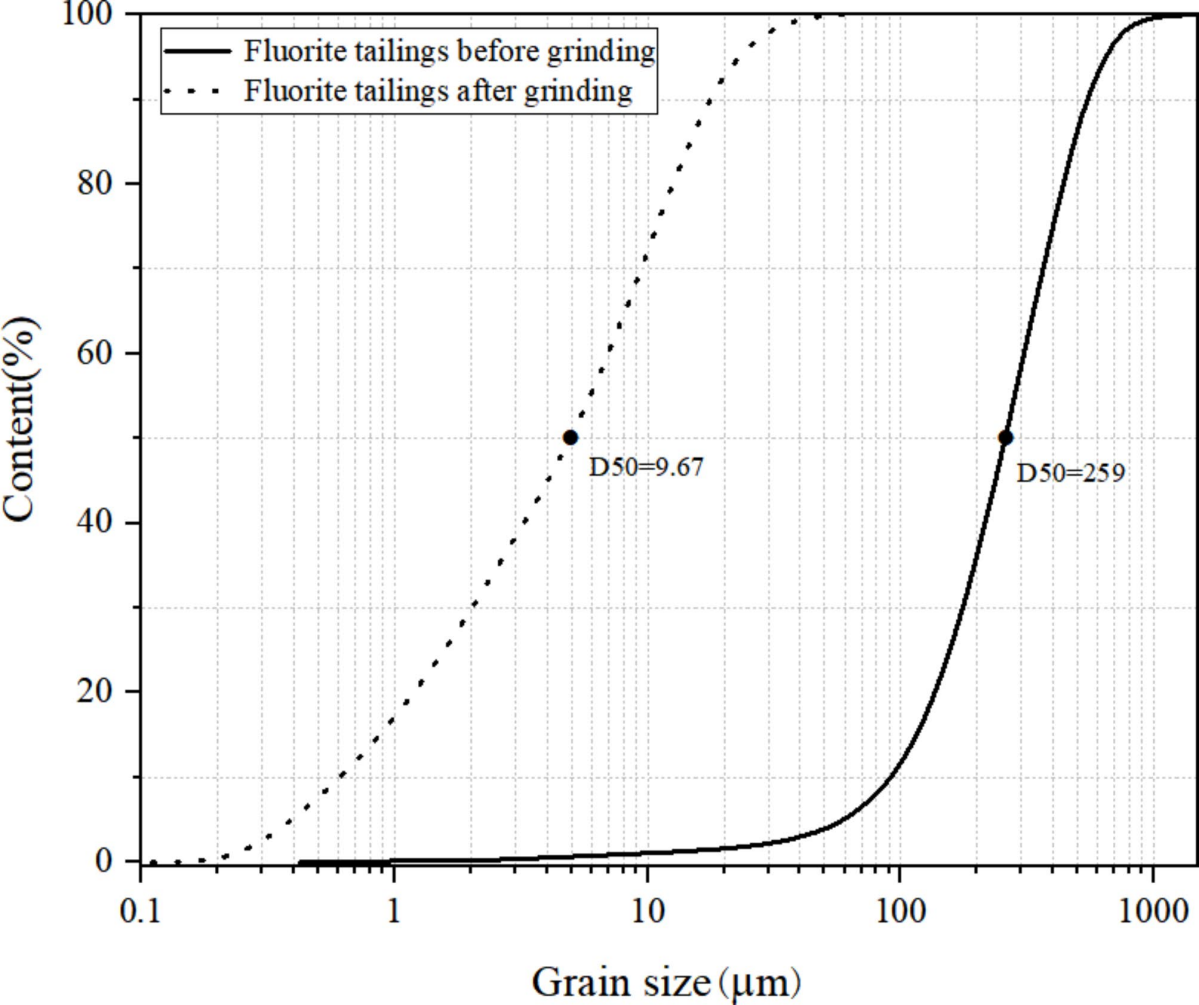
Laser particle size analysis of fluorite tailings.

**Fig. 3:**
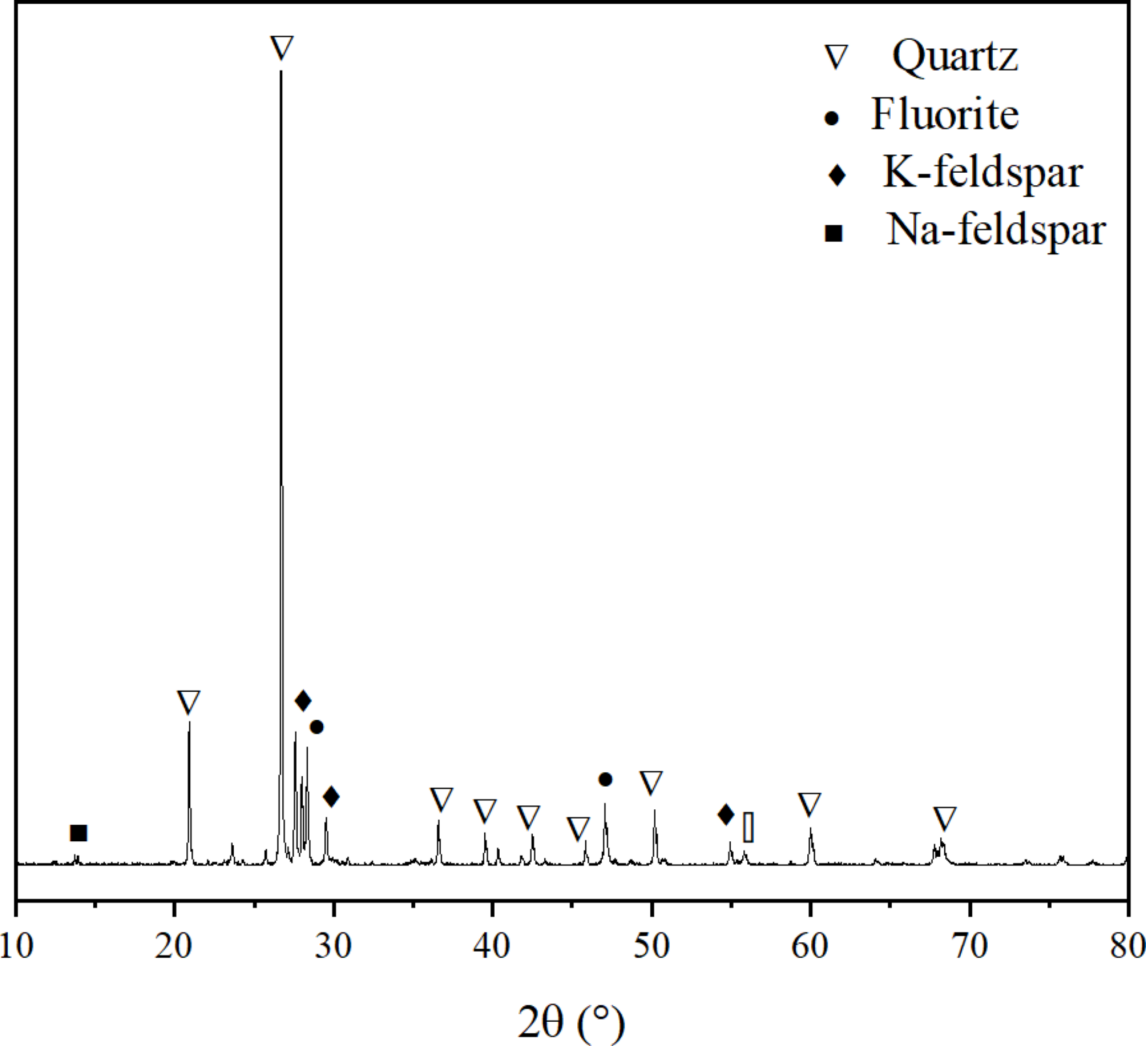
XRD analysis of fluorite tailing.

**Fig. 4:**
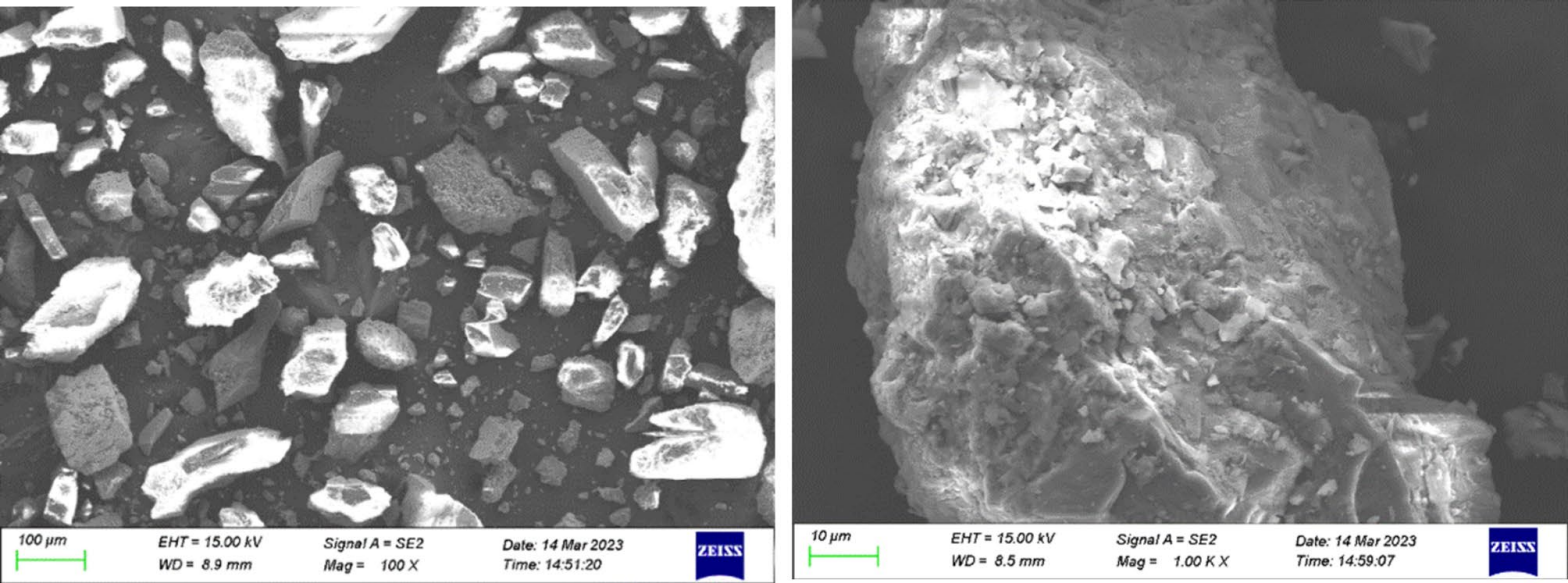
SEM morphology of fluorite tailings.

The fluorite tailings used in this experiment were obtained from a tailings pond in Luoyang as shown in Fig. 1 and their physical properties and chemical components are shown in Tables 1 and 2, whose Si/Al and Ca/Si ratios were 6.81 and 0.12. Laser particle size analysis of the fluorite tailings before and after grinding is shown in Fig. 2. The particle size range of the unpolished fluorite tailings was 300 μm, and D50 was 259 μm. After grinding, it is mainly distributed at 10 μm, D50 is 9.67 μm. The particle size decreased significantly. The XRD and SEM results of fluorite tailings are shown in Figs. 3 and 4 which indicate that the mineral phases of fluorite tailings are mainly quartz and fluorite, and a certain amount of feldspar can be observed^31^. The particle morphology is angular, with some attachments on the surface.

**Table 1:** Physical property of fluorite tailings.

	Apparent density	Bulk density	Specific surface area
Fluorite tailings	2.60 g/cm^3^	1.29 g/cm^3^	22.83 m^2^/kg

**Table 2:** Chemical composition of fluorite tailings (by weight, %).

	SiO_2_	Fe_2_O_3_	Al_2_O_3_	K_2_O	CaO	Na_2_O	MgO	LOI
Fluorite tailings	66.29	1.92	9.74	4.5	7.92	0.65	0.35	2.15

## Method

The physical properties and chemical components of fluorite tailings were tested according to the National Standards of the People’s Republic of China GBT 17431.1–2010^32^ and GBT 17431.2–2010^33^, respectively.An SM-500 laboratory ball mill was used to grind the fluorite tailings for 30 min. The phase composition of the sample was measured and analyzed by SmartLab SE X-ray diffractometer produced by Rigaku Company in Japan. During the operation of the X-ray diffractometer, the test conditions were Cu target radiation, the set voltage and current of the optical tube were 40 KV and 40 mA respectively, the test scanning range was 5–80˚ (2θ), the step value was 0.01˚ (2θ), and the scanning speed was 20˚ (2θ) /min. The micromorphology of fluorite tailings was tested by Singa 300 scanning electron microscope. The fluorite tailings were put into an electric blast oven and dried at 65  °C until the constant weight. Before using scanning electron microscopy, the sample was sprayed with gold with the small ion sputtering instrument of SSBC-12 in order to better observe the microscopic morphology of fluorite tailings.

In this experiment, the NBD-M1700-30I T high temperature sintering furnace produced by Norbadi Material Technology Co., Ltd. was used to activate the fluorite tailings. The basic activators used for active activation were analytically pure NaOH and Na_**2**_CO_**3**_, and the calcining temperatures were selected at 850 °C, 1000 °C and 1150 °C, respectively. The heating rate is 5 °C/min, and the holding time is 30 min. After the grinding fluorite tailings and geopolymerization are evenly mixed together using sieve mixing method according to the ratio in Table 3, the dealt tailings are calcined in a high-temperature sintering furnace, cooled to room temperature, and ground to less than 80 μm. XRD diffraction analysis, SEM and EDS analysis are carried out to evaluate the activation effect.

**Table 3:** Experimental ratio of alkali thermal activation.

Number	Calcination temperature/ °C	Chemical reagent/wt,%		Paste number
		NaOH	Na_2_CO_3_	
H20T850	850	20	0	PH20T850
H50T850	850	50	0	PH50T850
C20T850	850	0	20	PC20T850
C50T850	850	0	50	PC50T850
H20T1000	1 000	20	0	PH20T1000
H50T1000	1 000	50	0	PH50T1000
C20T1000	1 000	0	20	PC20T1000
C50T1000	1 000	0	50	PC50T1000
H20T1150	1 150	20	0	PH20T1150
H50T1150	1 150	50	0	PH50T1150

**Fig. 5:**
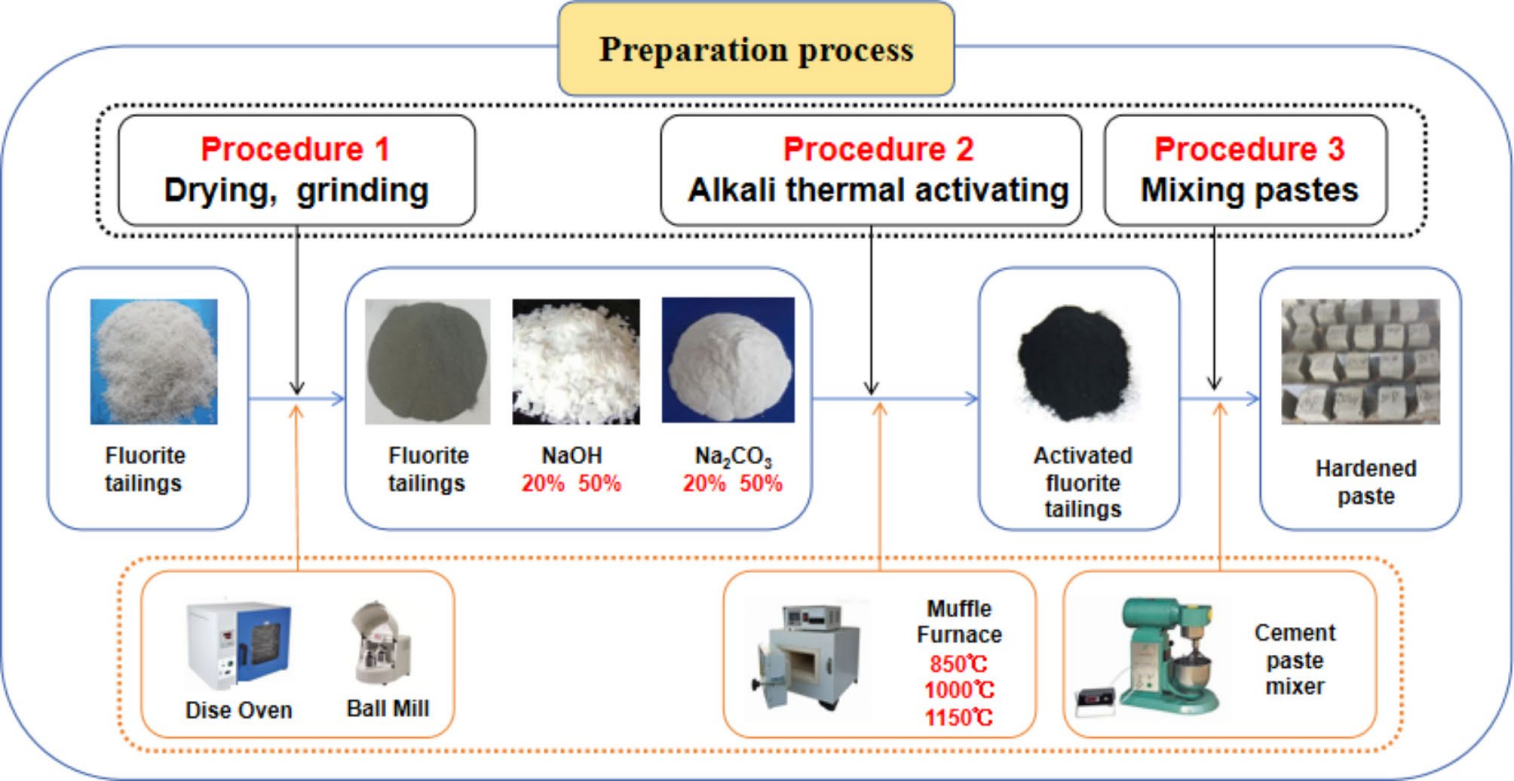
Pulp test block preparation.

Pastes were made of fluorite tailings under different alkaline heat excitation conditions, and the corresponding numbers are listed in Table 3. The water-binder ratio of the molding was 0.3, and the size of the test block was 20 mm×20 mm×20 mm. After molding, the test block was sealed with a plastic wrap and maintained at room temperature. The flow chart of Pulp test block preparation is shown in Fig. 5. After curing for 28d and 56d, the compressive strength was measured using a DY-208JX integrated folding and compression machine, which was tested according to the National Standards of the People’s Republic of China GB/T 17,671 − 2021, and the hydration products were analyzed by XRD, FTIR infrared spectroscopy, and SEM micromorphology.

## Results and discussion

### Analysis of activation effect of alkali heat activation of fluorite tailings

**Fig. 6:**
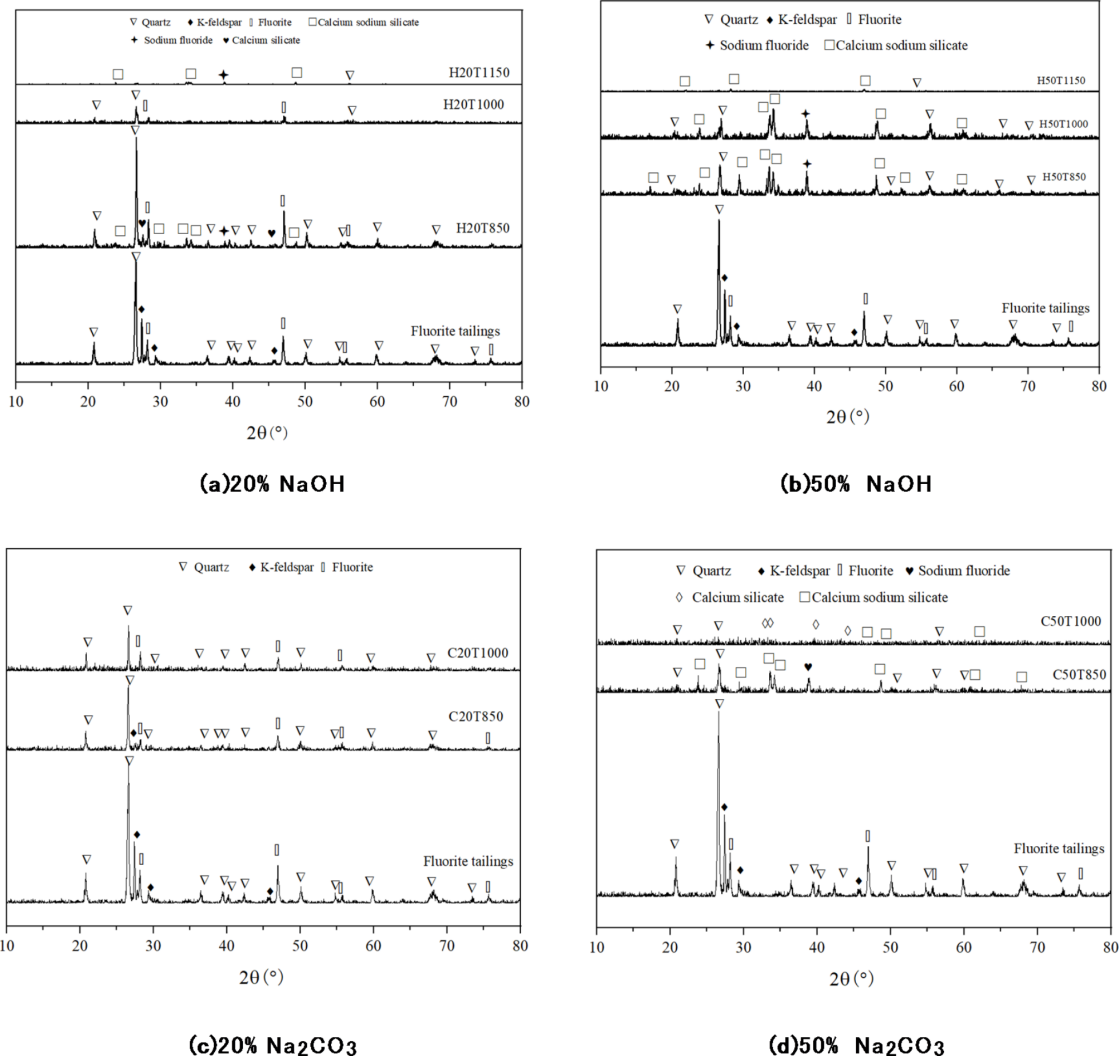
XRD diffraction pattern of fluorite tailings under different activation conditions.

Figure 6 shows the XRD diffraction patterns of different amounts and types of alkali thermally activated fluorite tailings at different temperatures, which are compared with those of unactivated fluorite tailings.

**Fig. 7:**
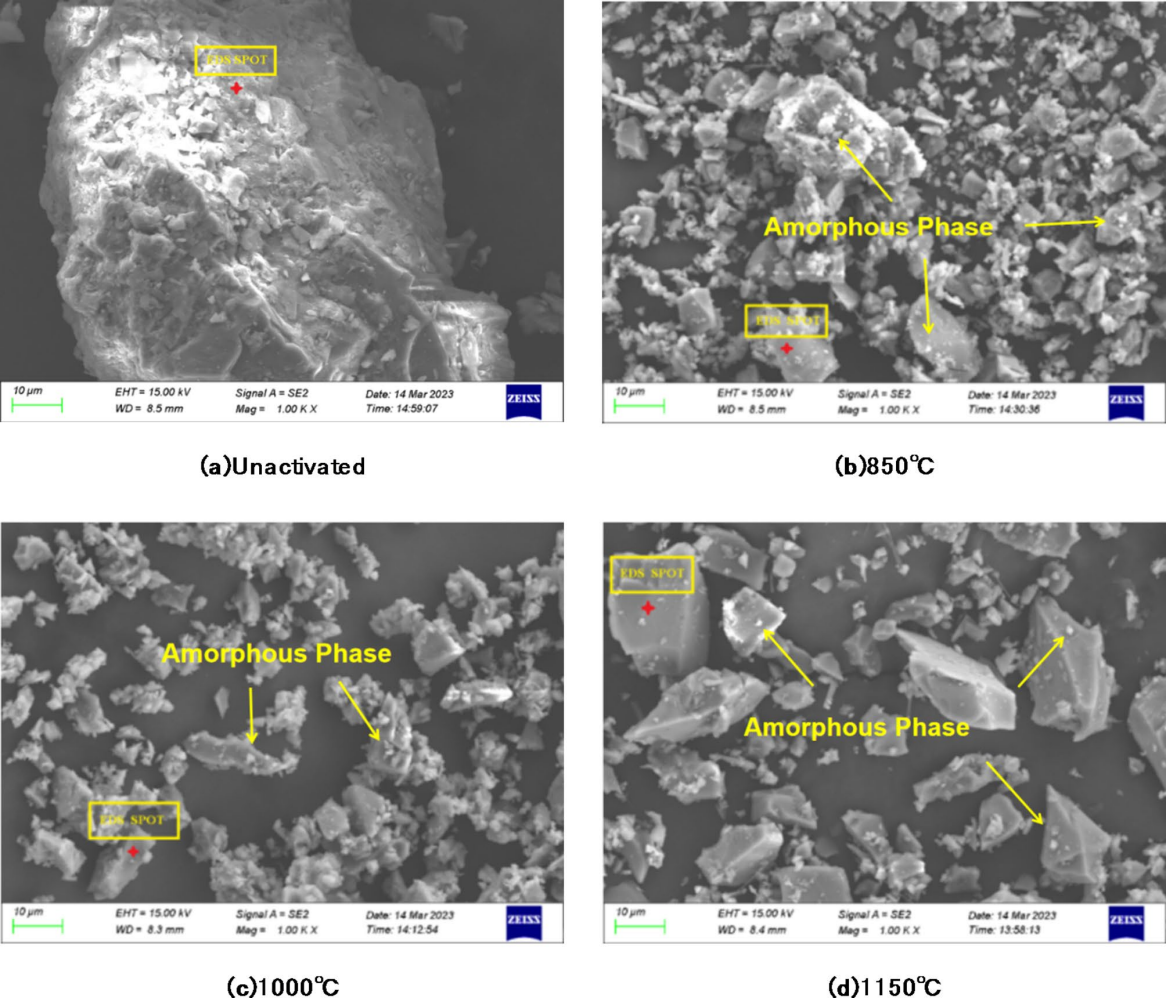
SEM of fluorite tailings at different temperatures under the condition of 50%NaOH alkali thermal activation.

For the fluorite tailings under the condition of NaOH activation, the original diffraction peak gradually disappears with the gradual increase of heat treatment temperature. When the activation temperature reaches 1000 °C, a large number of amorphous glass phases can be observed in the samples activated by 20% and 50%NaOH, and the latter one is more obvious in the alkali activation effect, while the amorphous phase decreases when the temperature rises to 1150 °C. Amorphous glass phase increases gradually with the rise of temperature, but when the temperature is too high, liquid phase appears in the Sodium-Silicon-Aluminum system, which reduces the amount of amorphous glass phase. For fluorite tailings activated by Na_2_CO_**3**_, when 20%Na_2_CO_3_ is used for activation, the difference is not obvious at different temperatures, while amorphous phase appears when 50%Na_2_CO_3_ activation reaches 1000 °C, indicating that the activation effect of Na_**2**_CO_3_ under high temperature treatment is weaker than that of NaOH. Through the comparative analysis of XRD diffraction pattern, it can be seen that the thermal activation effect of using NaOH alkali is better than Na_2_CO_**3**_, and the effect is relatively best when the activation temperature is 1000 °C, and the amorphous form decreases when the temperature continues to rise to 1150 °C, which may reduce the reactivity. Therefore, the difference of fluorite tailings under 50%NaOH alkaline thermal activation was further observed by SEM morphology and EDS spectrum analysis under different activation temperatures which shown in Fig. 7; Table 4.

**Table 4:** EDS of spot scanning of fluorite tailings under different activation conditions (by weight, %).

Element	Element composition						
	O	Na	Al	Si	K	Ca	Fe
Unactivated fluorite tailings	69.6	0.11	1.46	26.11	0.24	0.45	3.84
H50T850	49.92	24.54	3.06	20.07	1.31	0.58	0.52
H50T1000	54.67	13.58	3.12	22.79	2.02	3.27	0.55
H50T1150	61.17	8.71	2.60	20.70	1.43	4.74	0.65

It can be seen from the morphology observation that when NaOH is added to the fluorite tailings, it can be observed that the surface adhesion or amorphous phase is produced at high temperature. Combined with energy spectrum analysis, the amorphous phase may be the amorphous albite produced by the activation of crystalline albite in the tailings. When the activation temperature reaches 1150 °C, the surface of the particles becomes smoother and denser, indicating that a dense glass phase may be produced at high temperature, which is not conducive to its hydration reactivity.

### Analysis of hydration properties of fluorite tailings stimulated by alkali heat

**Fig. 8:**
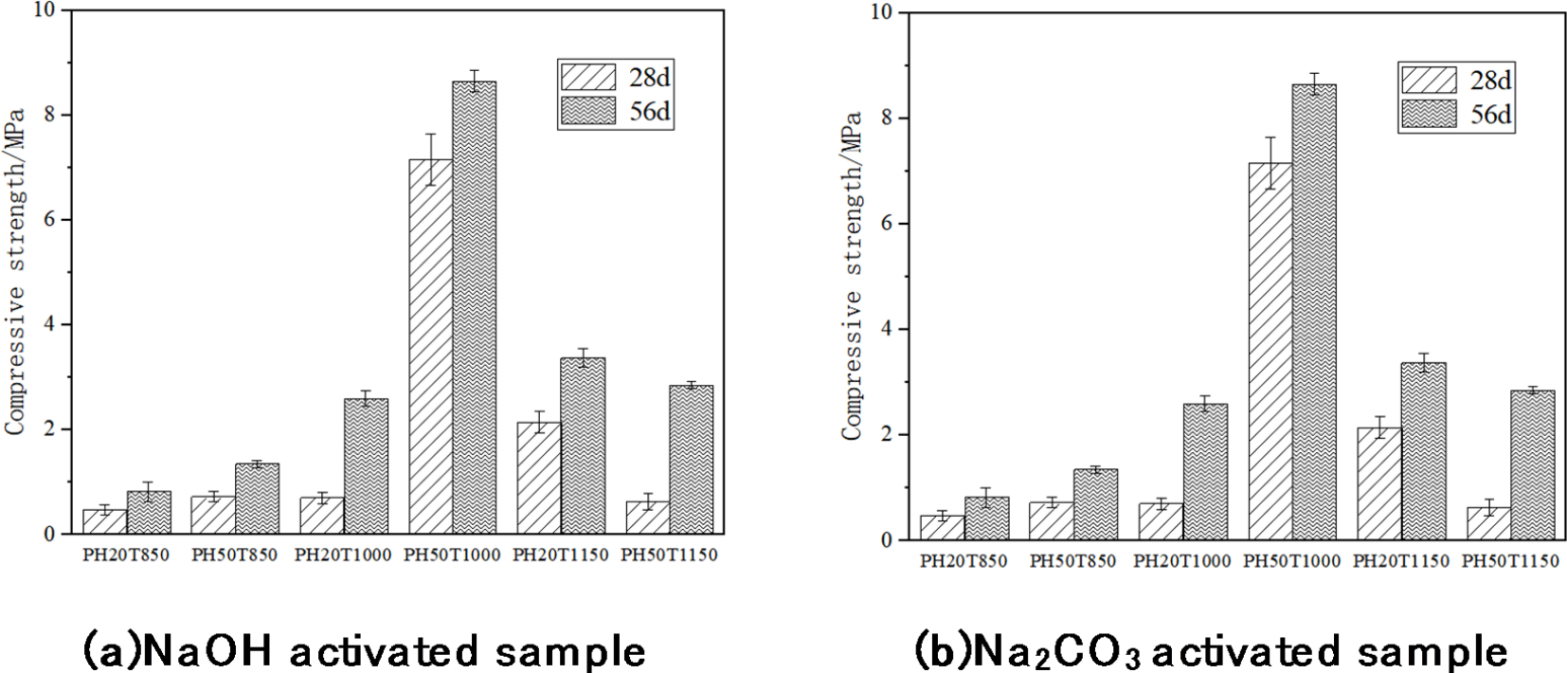
Compressive strength of fluorite tailings hardened paste under alkaline heat activation.

Figure 8 shows the compressive strength test data at 28d and 56d of the hardened paste prepared by alkaline heat activation of fluorite tailings.

It can be seen from the compressive strength test data that fluorite tailings under alkaline heat activation can exhibit a certain strength after reacting with water. The strength of the hardened paste under the activation conditions of NaOH at 1000 °C can reach more than 8 MPa at 56d, which is suitable for use as a geopolymer precursor. Under the activation condition of 850 °C, the strength of each sample was below 1 MPa, indicating that the activation condition of the fluorite tailings was relatively insufficient at this temperature. At 850 °C and 1000 °C, more NaOH and higher temperature activated the fluorite tailings better and increased the compressive strength, but at 1150  °C, the compressive strength did not increase, proving that too high temperature led to a decline in the activation effect. Compared with NaOH, Na_2_CO_3_ shows a lower intensity at the same activation temperature, and the maximum intensity can reach approximately 3 MPa at 56d at 1000 °C, indicating that NaOH has a relatively good effect on alkali thermal activation. At an activation temperature of 1000 °C, the strengths of the PH20T1000 and PC20T1000 samples with a small amount of alkali activator increased significantly in the later period. The compressive strength of the PH20T1000 sample at 56d d was approximately three times that at 28d, indicating that when 20%NaOH was used for activation, a longer prolonged hydration time resulted in more complete hydration of the specimen, more hydration products were generated, and a denser structure was formed, resulting in a higher compressive strength. The strength of the PH20T1000 sample could reach three times that at 28d. It has reactive activity at 1000 °C, but its initial activity is low.

**Fig. 9:**
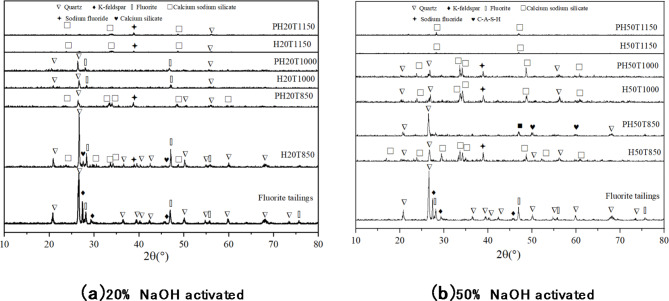
XRD analysis of fluorite tailings hydration products after alkali heat activation.

Based on the compressive strength test, XRD analysis was carried out on the sample of NaOH alkali thermal activation fluorite tailings for 28d hydration, the hydration products of fluorite tailings under different alkali thermal activation conditions were analyzed, and the XRD patterns are shown in Fig. 9.

As can be seen from the figure, for the hydration process of fluorite tailings activated by NaOH at different temperatures, the crystalline diffraction peaks of the samples activated at 850 °C and 1150 °C are obvious, while the peaks of amorphous phase are weaker than sample activated at 1050 °C. From the comparison between XRD pattens before and after 28d hydration, the amorphous phase in fluorite tailings hydrated in the curing period and more amorphous hydration products are observed.

**Fig. 10:**
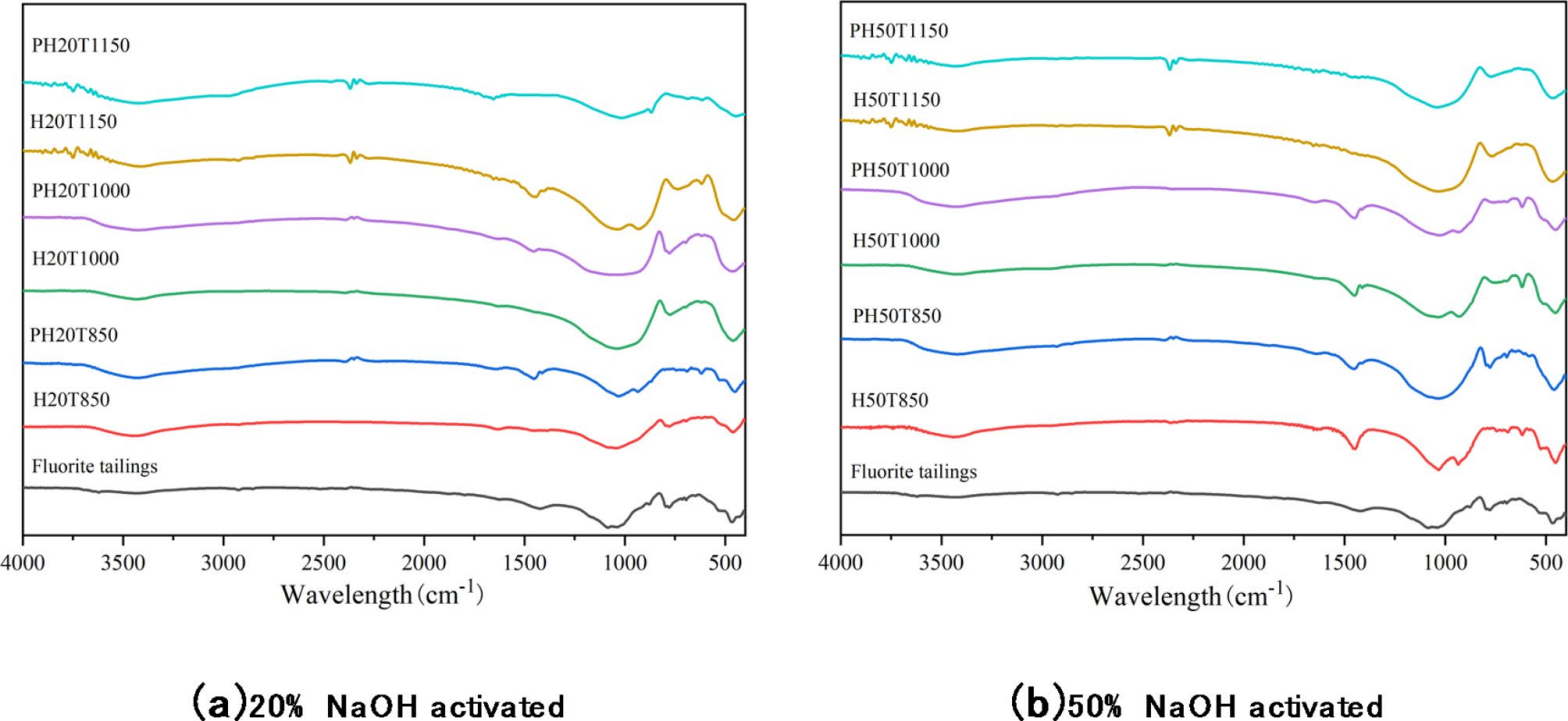
FTIR analysis of fluorite tailings hydration products activated by NaOH alkali heat.

Figure 10 shows FTIR analysis of hydration products of fluorite tailings stimulated by NaOH alkali heat at different temperatures.

As shown in the Fig. 10, the wide infrared absorption band of 800–1300 cm-1 the fluorite tailings activated by NaOH alkali heat narrowed after hydration and moved to a higher wavelength region. A small asymmetric stretching band appeared in the 950–990 cm − 1 region. This indicates that more Si and Al in the fluorite tailings after alkali heat activation were in a free state, which promoted the depolymerization and repolymerization of Si and Al. High temperatures stimulate the activity of Si and Al monomers, and promote the formation of Si and Al polycrystals.

**Fig. 11:**
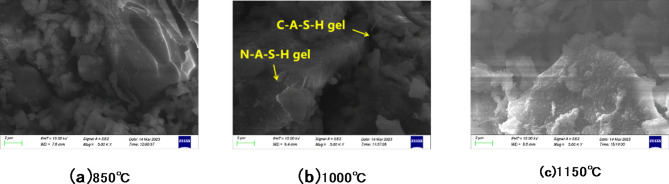
Hydration products of fluorite tailings under 50% sodium hydroxide alkaline heat activation.

Figure 11 shows the micro-morphology analysis of the hydration products in the hardened paste of fluorite tailings after 28 days of hydration under the condition of 50% sodium hydroxide alkaline heat activation.

According to the scanning electron microscope observation results, some active products are attached to the surface of the particles after alkali heat activation, and the surface hydration products of the fluorite tailings activated at 1000 °C can be clearly observed after hydration. Combined with the chemical composition and mineral phase analysis of the fluorite tailings, the hydration product may be the coexistence of amorphous N-A-S-H gel and a relatively structured C-A-S-H gel. On the other hand, the surface hydration products of fluorite tailings activated at 850 °C and 1150 °C were lower, thus showing lower strength.

## Conclusion

- XRD proves that under alkaline thermal excitation, a large number of amorphous phases are formed in the fluorite tailings, and the reactivity is significantly improved. The best thermal excitation temperature is 1000 °C, and there is not enough amorphous glass phase for hydration reaction at lower or higher temperatures.
- The mechanical properties of the test block proved that the fluorite tailings were excited by alkali heat at 1000 °C with 50% sodium hydroxide, and the compressive strength of the sample hardened 28 days after excitation reached 7.2 MPa, showing good reactivity.

Fluorite tailings have certain reactivity under alkaline thermal activation conditions and are feasible as geopolymer precursors. Although the method in the experiment is too idealized and expensive, it can provide guidance for the application of fluorite tailings.

## Data Availability

All data generated or analysed during this study are included in this published article.

## References

[1] Bei, B. H. et al. Fluorite deposits in China: Geological features, metallogenic regularity, and research progress. *China. Geol.* **3**(3), 473–489 (2020).

[2] Helbig, C., Baldauf, H., Mahnke, J., Stöckelhuber, K. W. & Schulze, H. J. Investigation of Langmuir monofilms and flotation experi-ments with anionic/cationic collector mixtures. *Int. J. Miner. Process.* **53**, 135–144 (1998).

[3] Chen, J. X., Yan, B. J., Li, H. W., Li, P., Guo, H. W. Vitrification of blast furnace slag and fluorite tailings for giving diopside-fluorapatite glass-ceramics. *Materials. Lett.* 218(2018).

[4] Lubisi, T. P., Nheta, W. & Ntuli, F. Physical, chemical and mineralogical characterization of fluorspar flotation tailings. *Mater. Today Proc.* **5**(1), 302–310. 10.1016/j.matpr.2017.11.086 (2018).

[5] Zhao, W. et al. Recycling of blast furnace slag and fluorite tailings into diopside-based glass-ceramics with various nucleating agents’ addition. *Sustainability* **13**(20), 11144. 10.3390/su132011144 (2021).

[6] Li, H. et al. Sintered glass-ceramic foams from fluorite tailings and waste glass with calcium phosphate addition. *Constr. Build. Mater.* **359**, 129528. 10.1016/j.conbuildmat.2022.129528 (2022).

[7] Mukherjee, D. P. & Das, S. K. SiO_2_–Al_2_O_3_–CaO glass-ceramics: Effects of CaF2on crystallization, microstructure and properties. *Ceram. Int.* **39**, 571–578. 10.1016/j.ceramint.2012.06.066 (2013).

[8] Rao, F. & Liu, Q. Geopolymerization and its potential application in mine tailings consolidation: A Review. *Miner. Process. Extr. Metall. Rev.* **36**, 399–409. 10.1080/08827508.2015.1055625 (2015).

[9] Provis, J. L. & van Deventer, J. S. J. *Alkali Activated Materials* (Springer, 2014). 10.1016/j.cemconres.2017.02.009.

[10] Ouffa, N., Benzaazoua, M., Belem, T., Trauchessec, R. & Lecomte, A. Alkaline dissolution potential of aluminosilicate minerals for the geosynthesis of mine paste backfill. *Mater. Today Commun.* **24**, 10.1016/j.mtcomm.2020.101221 (2020).

[11] Feng, D., Provis, J. L. & Deventer, J. S. J. V. Thermal activation of albite for the synthesis of one-part mix geopolymers. *J. Am. Ceram. Soc.* **95**(2012), 565–572. 10.1111/j.1551-2916.2011.04925.x (2012).

[12] Ke, X. et al. One-part geopolymers based on thermally treated red mud/NaOH blends. *J Am Ceram Soc* **98**, 5–11. 10.1111/jace.13231 (2015).

[13] Kiventerä, J. et al. Utilization of sulphidic tailings from gold mine as a raw material in geopolymerization. *Int. J. Miner. Process.* **149**, 104–110. 10.1016/j.minpro.2016.02.012 (2016).

[14] Kiventerä, J. et al. Alkali activation as new option for gold mine tailings inertization. *J. Clean. Prod.* **187**, 76–84. 10.1016/j.jclepro.2018.03.182 (2018).

[15] Koloušek, D. et al. Preparation, structure and hydrothermal stability of alternative (sodium silicate-free) geopolymers. *J. Mater. Sci.* **42**, 9267–9275. 10.1007/s10853-007-1910-5 (2007).

[16] Kovtun, M., Kearsley, E. P. & Shekhovtsova, J. Chemical acceleration of a neutral granulated blast-furnace slag activated by sodium carbonate. *Cement Concr. Res.* **72**, 1–9. 10.1016/j.cemconres.2015.02.014 (2015).

[17] Luukkonen, T., Abdollahnejad, Z., Yliniemi, J., Kinnunen, P. & Illikainen, M. One-part alkali-activated materials: a review. *Cement Concr. Res.* **103**, 21–34. 10.1016/j.cemconres.2017.10.001 (2018).

[18] Zhao, M. X. et al. Freeze-thaw durability of red mud slurry-class F fly ash-based geopolymer: Effect of curing conditions. *Constr. Build. Mater.* **215**, 381–390. 10.1016/j.conbuildmat.2019.04.235 (2019).

[19] Guo, X. L., Shi, H. S. & Dick, W. A. Compressive strength and microstructural characteristics of class C fly ash geopolymer. *Cem. Concr. Comp.* **32**(2), 142–147. 10.1016/j.cemconcomp.2009.11.003 (2010).

[20] Zhang, M. et al. Durability of red mud-fly ash based geopolymer and leaching behavior of heavy metals in sulfuric acid solutions and deionized water. *Constr. Build. Mater.* **124**, 373–382. 10.1016/j.conbuildmat.2016.07.108 (2016).

[21] McLellan, B. C., Williams, R. P., Lay, J., van Riessen, A. & Corder, G. D. Costs and carbon emissions for geopolymer pastes in comparison to ordinary portland cement. *J. Clean. Prod.* **19**(9–10), 1080–1090 (2011).

[22] Xu, Z. H. et al. Immobilization of strontium-loaded zeolite A by metakaolin based-geopolymer. *Ceram. Int.* **43**(5), 4434–4439. 10.1016/j.ceramint.2016.12.092 (2017).

[23] Juenger, M. C. G., Winnefeld, F., Provis, J. L. & Ideker, J. H. Advances in alternative cementitious binders. *Cem. Concr. Res.* **41**(12), 1232–1243. 10.1016/j.cemconres.2010.11.012 (2011).

[24] Zhang, X. L., Zhang, S. Y., Liu, H. & Zhao, Y. L. Disposal of mine tailings via geopolymerization. *J. Clean. Prod.* **284**, 124756. 10.1016/j.jclepro.2020.124756 (2021).

[25] Moukannaa, S. et al. Alkaline fused phosphate mine tailings for geopolymer mortar synthesis: Thermal stability, mechanical and microstructural properties. *J Non-Cryst Solids* **511**, 76–85. 10.1016/j.jnoncrysol.2018.12.031 (2019).

[26] Luo, Y. P., Bao, S. X. & Zhang, Y. M. Preparation of one-part geopolymeric precursors using vanadium tailing by thermal activation. *J. Am. Ceram. Soc.* **103**, 1–5. 10.1111/jace.16835 (2020).

[27] Ye, N. et al. Synthesis and strength optimization of one-part geopolymer based on red mud. *Constr. Build. Mater.* **111**, 317–325. 10.1016/j.conbuildmat.2016.02.099 (2016).

[28] Peng, M. X., Wang, Z. H., Shen, S. H., Xiao, Q. G. & Hu, L. L. Alkali fusion of bentonite to synthesize one-part geopolymeric cements cured at elevated temperature by comparison with two-part ones. *Constr. Build. Mater.* **130**, 103–11216. 10.1016/j.conbuildmat.2016.11.010 (2016).

[29] Liu, Q., Li, X. C., Cui, M. Y., Wang, J. X. & Lyu, X. J. Preparation of eco-friendly one-part geopolymers from gold mine tailings by alkaline hydrothermal activation. *J. Clean. Prod.* **289**, 126806. 10.1016/j.jclepro.2021.126806 (2021).

[30] Liu, Q., Sun, S. K., Zhang, J. K., Wan, J. X. & Lyu, X. J. Effect of CaO on hydration properties of one-part alkali-activated material prepared from tailings through alkaline hydrothermal activation. *Constr. Build. Mater.* **308**, 124931. 10.1016/j.conbuildmat.2021.124931 (2021).

[31] Hongwei, Li. et al. Sintered glass-ceramic foams from fluorite tailings and waste glass with calcium phosphate addition. *Constr. Build. Mater.*10.1016/j.conbuildmat.2022.129528 (2022).

[32] GBT 17431.1-2010, Lightweight aggregates and its test methods—Part1: Lightweight aggregates.

[33] GBT 17431.2-2010, Lightweight aggregates and its test methods—Part 2: Test methods for lightweight aggregates.

